# A comparison between Lynch syndrome and sporadic colorectal cancer survivors’ satisfaction with their healthcare providers

**DOI:** 10.1002/cam4.1033

**Published:** 2017-02-17

**Authors:** Allison M. Burton‐Chase, Wendy M. Parker, Katrina M. Polivka, Ellen R. Gritz, Christopher I. Amos, Karen H. Lu, Patrick M. Lynch, Miguel A. Rodriguez‐Bigas, Y. Nancy You, Susan K. Peterson

**Affiliations:** ^1^ Department of Population Health SciencesAlbany College of Pharmacy and Health SciencesAlbanyNew York; ^2^ Division of Health Promotion & Behavioral ScienceUniversity of Texas Health Science Center‐Houston School of Public HealthHoustonTexas; ^3^ Department of Behavioral ScienceThe University of Texas MD Anderson Cancer CenterHoustonTexas; ^4^ Department of Community and Family MedicineDartmouth CollegeHanoverNew Hampshire; ^5^ Department of Gynecologic Oncology & Reproductive MedicineThe University of Texas MD Anderson Cancer CenterHoustonTexas; ^6^ Department of GastroenterologyHepatology& NutritionThe University of Texas MD Anderson Cancer CenterHoustonTexas; ^7^ Department of Surgical OncologyThe University of Texas MD Anderson Cancer CenterHoustonTexas

**Keywords:** Colorectal cancer, Lynch syndrome, provider communication, provider satisfaction, provider trust, sporadic cancer

## Abstract

This study evaluated provider satisfaction in a sample of colorectal cancer (CRC) survivors with and without Lynch syndrome (LS). Participants were case–case‐matched CRC survivors with (*n* = 75) or without (*n* = 75) LS (mean age of 55; range: 27–93). Participants completed a mailed questionnaire assessing demographics, clinical characteristics, healthcare utilization, psychosocial variables, and provider satisfaction. LS CRC survivors reported lower provider satisfaction scores on three subscales of the Primary Care Assessment Survey: communication (78.14 vs. 83.96; *P* < 0.05), interpersonal treatment (78.58 vs. 85.30; *P* < 0.05), and knowledge of the patient (60.34 vs. 69.86; *P* < 0.01). Among LS CRC survivors, predictors for mean communication and trust subscale scores were location of treatment and socioeconomic status. Higher mean depression scores also were associated with trust, while social support predicted higher satisfaction with communication. Sporadic CRC survivor satisfaction is driven largely by age (communication, interpersonal treatment) and patient anxiety (communication), while seeing a provider more often was associated with increased satisfaction with knowledge of the patient. LS CRC survivors reported lower levels of provider satisfaction than sporadic CRC survivors. LS survivors who received care at The University of Texas MD Anderson Cancer Center, a comprehensive cancer center (CCC), reported higher satisfaction than those receiving care at other institutions. Depressive symptoms and socioeconomic status may impact provider satisfaction ratings. Exploration of other potential predictors of provider satisfaction should be examined in this population. Additionally, further research is needed to examine the potential impact of provider satisfaction on adherence to medical recommendations in LS CRC survivors, particularly those being treated outside of CCCs.

## Introduction

Patients diagnosed with cancer often have increased healthcare needs, which may result in higher expectations of care. A greater understanding of predictors of patient satisfaction with healthcare providers (HCPs) in an oncology setting can provide valuable information to providers as well as researchers. There also may be differences between patients with sporadic colorectal cancer and those with an inherited syndrome such as Lynch syndrome. There is a significant gap in knowledge regarding the patient–provider relationship in the oncology literature generally and in the Lynch syndrome (LS) population specifically. By understanding the predictors of patient satisfaction with their HCP for individuals with LS, we can better inform community health providers, develop more targeted interventions, and ultimately improve screening and surveillance adherence. This study evaluates satisfaction with HCPs in a matched sample of LS and sporadic CRC survivors.

### What is LS?

LS is the most common hereditary colon cancer, accounting for approximately 3% of all colorectal cancer (CRC) cases 1. LS is characterized by predisposition to several cancers, most commonly CRC and endometrial cancer, and is caused by germline mutations in DNA mismatch repair (MMR) genes, specifically *MLH1, MSH2, MSH6, PMS2*, or in the *EPCAM* gene 2, 3, 4, 5, 6, 7. These mutations can be identified through clinical genetic testing, ideally initiated in individuals with cancer 8.

Compared with the general population, MMR mutation carriers have a higher lifetime risk for several cancers; CRC risk ranges from 20 to 69% for men and 10 to 52% for women, with risks varying based on mutated gene 9, 10, 11, 12, 13. Women with LS also have a 40–60% lifetime risk for developing endometrial cancer 2, 3, 8, 14. Individuals with LS are at increased risk for other malignancies, including ovarian, stomach, small bowel, hepatobiliary tract, pancreatic, urinary tract, brain, and skin cancers 3, 8, 14. To mitigate these risks, affected individuals are advised to follow high‐risk surveillance and cancer management guidelines 8, 15. Screening recommendations include annual or biennial colonoscopy initiated at age 20–25 years or 2–5 years younger than the earliest known case in the family, which has proven clinical benefits, and annual endometrial screening initiated at age 30–35 years. In women who have completed childbearing, prophylactic endometrial surgery also is recommended 3, 4, 8, 15.

### Patient–provider relationships in oncology settings

Patients’ satisfaction with their healthcare providers (HCPs) has been shown to be positively correlated with adherence to screening, surveillance, and treatment 16, 17. Improving adherence to screening for LS individuals is particularly important, as their screening regimens are demanding and nonadherence can be life‐threatening 16, 17. One important aspect of patients’ HCP satisfaction is the nature of the patient–provider relationship 16, 17, 18, 19, 20. Prior research on CRC survivors’ satisfaction with HCP has focused on treatment satisfaction or healthcare service quality, whereas little is known about characteristics of the patient–provider relationship, nor how such characteristics influence patient satisfaction in this population 16, 21, 22, 23.

For individuals with LS, one critical factor that impacts a HCP's ability to appropriately counsel patients with LS is a significant gap in knowledge about genetics and LS. HCP not only need to be adequately informed about the characteristics of LS, but also need to be able to obtain comprehensive medical and family histories, make referrals to genetics services, and recommend appropriate screening and medical management 4, 24, 25, 26, 27, 28, 29. Data from prior studies show that physicians lack knowledge in these key areas. One study focusing on gastroenterologists found that when presented with a family history consistent with LS, 79% of physicians could identify the syndrome, 26% recommended genetic counseling for the patient, and only 16% advised appropriate screening 30. In a study that compared knowledge of LS among a sample of gastroenterologists and primary care physicians, findings showed that gastroenterologists were more likely to elicit a family history of colorectal neoplasia (93% vs. 63%), implement appropriate screening strategies for individuals with LS (73% vs. 50%), and refer a patient at risk for LS for genetic testing (72% vs. 57%) 29. However, both groups of physicians demonstrated less‐than‐optimal compliance with recommended screening guidelines and with the notification of at‐risk relatives 29. HCP knowledge in these areas is key in the LS population as research has indicated that simply informing an individual of his or her mutation status and cancer risk may not motivate behavior change, and may, in fact, be a barrier to screening if the individual believes that they have no control over whether he or she develops cancer 31, 32, 33.

A variety of tools have been used to evaluate patient satisfaction with their HCPs in the general population, but few have been used in oncology settings 34. The variation in measures and populations used to assess patient satisfaction with HCP makes it difficult to compare satisfaction results across studies. Thus, the literature examining the patient–provider relationship in oncology, and how it relates to satisfaction, remains limited 34. To more clearly articulate the patient–provider relationship in oncology and LS populations, we must first understand the predictors of patient satisfaction with their HCP 21, 34.

Several studies have identified trust and physician knowledge as important predictors of patient satisfaction with HCP in oncology settings 14, 17, 19, 34. One study, focusing specifically on LS mutation carriers, examined barriers and facilitators of screening and management in this population 14. Both trust and physician knowledge of family history were interdependent factors that affected LS patients’ perception of their HCPs. Study participants expressed higher levels of trust when physicians were both aware of their family history and recognized the importance of high‐risk cancer surveillance 14. These factors also influenced participation in recommended screening regimens 14. However, further research is needed to fully examine how patient trust can be effectively improved 17.

Other relationship factors, including communication and interpersonal behavior, also have been used to assess patient satisfaction with HCPs in oncology settings 16, 18, 20, 35. One study found that through effective communication, HCPs were able to positively impact health‐related attitudes and behaviors regarding adherence 18. A particularly salient finding from this study was that communication style was the only patient or HCP variable that could both influence screening adherence and be taught 18. In addition to improving patient satisfaction with their HCPs and health behaviors, patient–provider communication may be related to patients’ overall quality of life 16, 20. Providers’ interpersonal and socio‐emotional behaviors, such as empathy, engagement, and attentiveness, also appear to be predictors of patients’ perception of their HCPs 20.

The aim of this study was to evaluate satisfaction with HCPs in a matched sample of LS and sporadic CRC survivors. Directly comparing these two populations enabled us to identify factors related to provider satisfaction that may be unique to the LS population while also adding to the limited literature on patient satisfaction with their providers in an oncology setting.

## Materials and Methods

### Participants

This study was approved by the Institutional Review Board at the University of Texas MD Anderson Cancer Center (MD Anderson). Participants were CRC survivors with LS or sporadic cancer who were matched on age, sex, race/ethnicity, cancer stage, geography, and time since diagnosis using a LS case–sporadic case design. Survivors with LS were recruited from MD Anderson (*n* = 33) and through social media (*n* = 42) and had to have tested positive for a LS mutation. Sporadic CRC survivors were recruited from the tumor registry at MD Anderson (*n* = 75). All participants were 18 years of age or older and were able to read and speak English. Patients with CRC were limited to those with a diagnosis of CRC from 6 months to 5 years prior to enrolling in the study. LS participants recruited through MD Anderson were screened for eligibility using medical records and those recruited through social media were screened by self‐report over the phone prior to enrolling in the study. Using information from their medical records, we excluded sporadic CRC patients with a personal or family history of familial adenomatous polyposis (FAP) and inflammatory bowel disease, or those who had a first‐degree relative with CRC.

### Data collection methods

Data were collected using a mailed, self‐administered questionnaire. Eligible survivors received a packet containing an introductory letter, questionnaire, and a self‐addressed stamped return envelope. Nonrespondents received an identical follow‐up mailing at 3 weeks after the initial mailing and a follow‐up reminder phone call at 6 weeks with the option to complete the questionnaire over the phone. Those who completed the questionnaire received a $10 gift card as compensation.

### Study measures

Demographic data were obtained through self‐report. Medical data were obtained through medical records and self‐report.

The location of treatment for each patient was noted in an open‐ended question and then coded as an NCI‐designated comprehensive cancer center (CCC) or other healthcare institution. All individuals seen at a CCC received care at MD Anderson. Healthcare utilization was measured using a 4‐item scale developed by the Stanford Chronic Disease Self‐Management Study 36. This measure quantifies physician, emergency room, and hospital visits.

The 20‐item Center for Epidemiologic Studies Depression (CES‐D) scale measured depressive symptoms 37. A clinical psychologist was available for referral for individuals who were identified as showing high levels of depressive symptomatology. Anxiety was measured using the State‐Trait Anxiety Inventory (STAI). Both the CES‐D and STAI are widely used in clinical and medical populations and have good internal consistency and reliability 37, 38. Social support and satisfaction with social support were assessed using a scale by Krause and Borawski‐Clark 39.

We used four scales from the Primary Care Assessment Survey (PCAS) to measure the patient's relationship with the treating provider for our outcome, including communication, interpersonal treatment, patient trust, and provider's knowledge of the patient (comprehensive) 40, 41, 42. The instructions for the PCAS ask the patient to “think about the one healthcare provider who is most involved in coordinating his or her care” when responding to the questions. The measure does not specifically ask for provider specialties. In prior research, these scales have correlated well with outcomes of care such as adherence and satisfaction 40, 41, 42.

### Statistical analysis

Bivariate differences were evaluated between LS and sporadic survivors on known or expected factors related to provider satisfaction using chi‐square, and completed with SAS version 9.3 43. Regression models were estimated separately for LS and sporadic survivors to independently evaluate the factors that predicted provider satisfaction. Distinct models were estimated for each of the four PCAS subscales (communication, interpersonal treatment, trust, and provider's knowledge of the patient) in each group.

## Results

### Demographic and bivariate analysis

From the overall sample of 75 LS CRC and 75 sporadic CRC survivors, we excluded seven who were missing at least one component of our outcome measure, the PCAS scale. Our analytic sample for this study included 73 LS CRC and 70 sporadic CRC survivors. The average age of participants was in their early 50s with a mean age of 52.6 years for patients with LS and 54.1 for patients with sporadic cancer, with more females than males (54.9% and 57.1%, respectively) and LS more likely to be married (83.6% and 77.1%, respectively, for LS versus sporadic). Demographic characteristics can be seen in Table 1. Due to the matching of LS and sporadic cases, there were no significant demographic differences between groups. We also assessed differences between the LS CRC survivors who were recruited through MD Anderson and those recruited through social media and found no significant differences.

**Table 1:** Demographics *n* (%) [95% confidence interval]

Characteristic		LS (*n* = 73)	Sporadic (*n* = 70)	*P*‐value
Mean Age (SD)		52.6 (12.1) [49.8–55.5]	54.1 (11.3) [51.4–56.8]	(*P* = 0.46)
Gender	Female	40 (54.8%) [0.431–0.665]	40 (57.1%) [0.453–0.690]	(*P* = 0.78)
	Male	33 (45.2%) [0.335–0.569]	30 (42.9%) [0.310–0.547]	
Marital Status	Married	61 (83.6%) [0.749–0.923]	54 (77.1%) [0.671–0.872]	(*P* = 0.34)
	Not married	12 (16.4%) [0.078–0.252]	16 (22.9%) [0.128–0.329]	
Race	White	67 (91.8%) [0.853–0.982]	66 (94.3%) [0.887–0.999]	(*P* = 0.56)
	Non‐white	6 (8.2%) [0.018–0.147]	4 (5.7%) [0.001–0.113]	
Child	Have children	60 (82.2%) [0.732–0.912]	63 (90.0%) [0.828–0.972]	(*P* = 0.18)
	No children	13 (17.8%) [0.078–0.252]	7 (10.0%) [0.078–0.252]	
Work	Working full‐ or part‐time	46 (63.0%) [0.517–0.744]	42 (60.0%) [0.482–0.718]	(*P* = 0.71)
	Not working	27 (37.0%) [0.256–0.483]	28 (40.0%) [0.282–0.518]	
Education	Less than college	27 (37.0%) [0.256–0.483]	34 (48.6%) [0.366–0.606]	(*P* = 0.16)
	College degree	23 (31.5%) [0.206–0.424]	23 (32.9%) [0.216–0.441]	(*P* = 0.86)
	Postgraduate	23 (31.5%) [0.206–0.424]	13 (18.6%) [0.092–0.279]	(*P* = 0.08)
Financial Situation	Financial difficulty	17 (23.3%) [0.134–0.332]	15 (21.4%) [0.116–0.313]	(*P* = 0.79)
	No spare money	19 (26.0%) [0.157–0.363]	23 (32.9%) [0.216–0.441]	(*P* = 0.37)
	Can afford special things	37 (50.7%) [0.389–0.624]	32 (45.7%) [0.338–0.577]	(*P* = 0.56)

Not all percentages and totals add up due to missing data.

As illustrated in Table 2, healthcare utilization was similar between the LS and sporadic CRC groups. Within the last 6 months, most had seen their doctors just under four times and had less than one emergency room visit or hospital admission. There were no significant differences between LS and sporadic CRC survivors on any psychosocial measures (CES‐D, Trait, or State anxiety scales). Satisfaction with social support and family support showed no differences between the two groups. There was a significant difference in friend support, with LS CRC survivors scoring lower than sporadic CRC survivors (LS = 23.647; sporadic = 25.706; *P* = 0.03). There were no differences on any of these variables between the two recruitment sources for LS survivors.

**Table 2:** Healthcare experiences, social support, and patient satisfaction mean scores *n* (%) [95% confidence interval]

Mean/(SD)		LS (*n* = 73)	Sporadic (*n* = 70)	*P*‐value
Location of treatment	Comprehensive cancer centers	33 (45.2%) [0.335–0.569]	70 (100%)	c(*P* < 0.001)
	Noncomprehensive cancer centers	40 (54.8%) [0.431–0.665]	0 (0%)	
Healthcare experiences (Number)	Doctor visits (past 6 months)	3.834 (3.782) [2.951–4.716]	3.608 (2.870) [2.923–4.292]	(*P* = 0.69)
	Emergency room visits (past 6 months)	0.329 (1.334) [0.018–0.640]	0.200 (0.554) [0.068–0.332]	(*P* = 0.46)
	Different hospital stays (past 6 months)	0.219 (0.917) [0.005–0.433]	0.186 (0.460) [0.076–0.295]	(*P* = 0.78)
	Total overnight hospital stays (past 6 months)	0.930 (4.489) [‐0.117–1.978]	0.811 (2.529) [0.208–1.413]	(*P* = 0.85)
Psychosocial metrics	CES‐D Scale score	8.918 (7.496) [7.169–10.667]	9.298 (9.419) [7.052–11.544]	(*P* = 0.79)
	Trait score	66.023 (9.946) [63.702–68.345]	65.079 (12.778) [62.032–68.126]	(*P* = 0.62)
	State score	32.535 (8.308) [30.597–34.474]	33.112 (10.830) [30.528–35.693]	(*P* = 0.72)
Social support scales	Krause social support satisfaction scale	9.405 (2.162) [8.901–9.910]	9.779 (2.340) [0.005–0.433]	(*P* = 0.32)
	Lubben social support family scale	24.901 (4.973) [23.741–26.062]	25.762 (5.546) [24.440–27.084]	(*P* = 0.33)
	Lubben social support friend scale	23.647 (5.503) [22.363–24.931]	25.706 (5.688) [24.350–27.062]	a(*P* = 0.03)
PCAS scores	Communication	78.137 (18.120) [74.194–82.957]	83.957 (17.771) [74.194–82.957]	a(*P* = 0.05)
	Interpersonal	78.575 (18.778) [74.194–82.957]	85.429 (16.444) [81.508–89.349]	a(*P* = 0.02)
	Trust	76.795 (15.496) [73.179–80.410]	80.500 (13.663) [77.242–83.758]	(*P* = 0.13)
	Comprehensive	60.342 (21.071) [55.426–65.259]	69.714 (21.228) [64.653–74.776]	**(*P* = 0.01)

a *P* < 0.05.

b *P* < 0.01.

c *P* < 0.001.

Compared with sporadic CRC survivors, LS survivors reported lower mean provider satisfaction scores on three of the PCAS subscales: communication (78.14 vs. 83.96; *P* = 0.05), interpersonal treatment (78.58 vs. 85.30; *P* = 0.02), and physician's knowledge of the patient (60.34 vs. 69.86; *P* = 0.01). There was no statistical difference in provider trust between the two groups.

### Regression analysis

Tables 3 and 4 show the linear regression models for factors for each of the four PCAS subscales for LS and sporadic patients, respectively. Among LS CRC survivors, communication and trust subscales showed similar patterns of predictors. Having more education or having financial challenges was associated with increased mean scores for both communication and trust, while being treated outside of a CCC was associated with lower scores. Being treated at healthcare institution that was not a CCC was a predictor for lower mean scores on communication (−11.36; *P* < 0.01) and trust (−8.79; *P* < 0.05) subscales. Higher mean depression scores were associated with lower mean scores on the trust subscale (−0.84; *P* < 0.01). Higher mean scores on family support were associated with higher communication satisfaction scores (0.87; *P* < 0.05). There were no significant predictors for the interpersonal or comprehensive subscale measures.

**Table 3:** Regression model for patients with LS (*n* = 73)

Variable	Parameter estimate (SE)			
	Communication (*r* ^2^ = 0.458)	Interpersonal (*r* ^2^ = 0.374)	Trust (*r* ^2^ = 0.459)	Comprehensive (*r* ^2^ = 0.384)
Intercept	24.737 (23.710)	37.229 (27.569)	44.851 (21.149)	0.499 (30.675)
Age	0.304 (0.225)	0.245 (0.262)	0.184 (0.201)	0.365 (0.291)
Female	0.415 (4.382)	−0.835 (5.096)	−3.204 (3.909)	−2.248 (5.670)
Not Married	−11.130 (6.708)	−13.511 (7.799)	−9.746 (5.983)	−9.131 (8.678)
Child	−1.782 (6.013)	−1.170 (6.991)	1.527 (5.363)	6.270 (7.779)
Non‐white	7.453 (7.914)	3.210 (9.202)	−2.095 (7.059)	13.198 (10.239)
<College degree	7.293 (5.306)	4.005 (6.169)	6.882 (4.733)	6.443 (6.864)
Postgrad degree	11.879^a^ (5.124)	7.205 (5.958)	10.156^a^ (4.570)	11.956 (6.629)
Unemployed	0.750 (4.848)	−0.465 (5.637)	−4.079 (4.324)	−4.071 (6.272)
Financial difficulty	13.025^a^ (6.513)	6.256 (7.573)	13.145^a^ (5.810)	11.397 (8.426)
Moderate financial difficulty	2.874 (4.546)	1.415 (5.286)	5.564 (4.055)	8.328 (5.882)
# Dr. visits	0.253 (0.605)	0.589 (0.703)	0.250 (0.540)	−0.149 (0.783)
# ER visits	−3.996 (4.264)	−3.816 (4.958)	−4.133 (3.804)	0.823 (5.717)
# Hospital stays	6.778 (8.287)	11.628 (9.636)	6.813 (7.392)	15.531 (10.722)
# Hospital nights	−0.050 (1.771)	−1.141 (2.059)	−0.543 (1.580)	−3.236 (2.291)
CES‐D	−0.616 (0.323)	−0.664 (0.376)	−0.843^b^ (0.288)	−0.620 (0.418)
Krause	0.192 (0.652)	−0.330 (0.758)	−0.039 (0.582)	0.982 (0.843)
Lubben family	0.871^a^ (0.410)	0.810 (0.477)	0.698 (0.366)	0.959 (0.530)
Lubben friend	0.166 (0.410)	0.191 (0.477)	0.255 (0.366)	0.166 (0.530)
Trait	0.125 (0.131)	0.161 (0.152)	0.022 (0.117)	−0.004 (0.169)
State	−0.127 (0.218)	0.092 (0.253)	0.135 (0.194)	0.010 (0.282)
Non‐CCC	−11.361^b^ (4.061)	−7.709 (4.722)	−8.786^a^ (3.622)	−4.286 (5.254)

a *P* < 0.05.

b *P* < 0.01.

**Table 4:** Regression model for sporadic patients (*n* = 70)

Variable	Parameter estimate (SE)			
	Communication (*R* ^2^ = 0.283)	Interpersonal (*R* ^2^ = 0.312)	Trust (*R* ^2 ^= 0.152)	Comprehensive (*R* ^2 ^= 0.224)
Intercept	116.633 (29.030)	107.639 (26.328)	96.695^b^ (24.280)	34.036 (36.081)
Age	−0.588^a^ (0.266)	−0.545^a^ (0.242)	−0.389 (0.223)	−0.028 (0.331)
Female	−8.318 (5.052)	−6.081 (4.582)	−2.412 (4.225)	−1.658 (6.279)
Not Married	6.170 (7.008)	2.551 (6.356)	0.995 (5.861)	1.110 (8.710)
Child	6.497 (7.235)	7.081 (6.562)	3.059 (6.051)	7.171 (8.992)
Non‐white	−2.661 (10.098)	4.706 (9.158)	0.300 (8.445)	7.068 (12.550)
< college degree	4.865 (5.492)	3.323 (4.981)	2.909 (4.593)	10.420 (6.826)
Postgrad degree	−3.095 (6.873)	−4.464 (6.232)	1.415 (5.748)	1.454 (8.541)
Unemployed	3.646 (5.603)	5.826 (5.081)	3.807 (4.686)	3.804 (6.964)
Financial difficulty	1.214 (7.356)	1.613 (6.671)	−0.571 (6.152)	−3.804 (9.143)
Moderate financial difficulty	0.647 (5.954)	3.631 (5.340)	2.198 (4.980)	−5.389 (7.400)
# Dr. visits	1.705 (0.968)	1.339 (0.878)	0.567 (0.810)	2.451^a^ (1.203)
# ER visits	−3.907 (4.675)	−4.061 (4.240)	−2.969 (3.910)	2.101 (5.765)
# hospital stays	−0.467 (10.512)	3.783 (9.534)	2.485 (8.792)	−9.122 (13.066)
# hospital nights	0.602 (1.779)	0.283 (1.622)	0.052 (1.496)	1.845 (2.223)
CES‐D	0.315 (0.388)	0.187 (0.351)	−0.033 (0.324)	−0.091 (0.482)
Krause	0.867 (0.949)	1.365 (0.861)	0.544 (0.794)	−0.240 (1.179)
Lubben family	0.182 (0.433)	0.183 (0.393)	0.054 (0.362)	−0.119 (0.538)
Lubben friend	−0.525 (0.494)	−0.328 (0.448)	−0.182 (0.413)	0.773 (0.614)
Trait	0.058 (0.212)	−0.034 (0.193)	0.062 (0.178)	0.106 (0.264)
State	−0.570^a^ (0.286)	−0.448 (0.259)	−0.249 (0.239)	−0.082 (0.355)

a *P* < 0.05.

b *P* < 0.01.

Regression results for the sporadic CRC survivors differed from those of the LS survivors. There were no specific predictors for the trust subscale. Age was negatively associated with the communication and interpersonal subscale scores for sporadic survivors, indicating that as a patient gets older, he or she is less satisfied with providers in terms of communication and overall interaction (communication: −0.59; *P* < 0.05; interpersonal: −0.55; *P* < 0.05). Lower mean State anxiety scores were associated with higher satisfaction scores for these survivors (−0.57; *P* < 0.05). Increased utilization was associated with higher satisfaction with the comprehensive aspects of provider treatment (2.45; *P* < 0.05).

## Discussion

The goal of this study was to assess patient satisfaction with their healthcare providers in a sample of CRC survivors both with and without LS. Specifically, we were interested in comparing these two groups and then assessing factors drawn from the literature that impact these ratings of HCP satisfaction. Having a better understanding of these complex patient–provider relationships can both inform community health providers and assist in the development of future interventions.

In our study, LS CRC survivors reported lower levels of satisfaction with their healthcare providers than sporadic CRC survivors, especially as related to communication, interpersonal interactions, and knowledge of the patient as a person. There were no differences between the two groups in our study in terms of ratings of provider trust. LS CRC survivors who received care at CCCs reported higher satisfaction when compared to those receiving care at other institutions, specifically in terms of communication and trust. Depressive symptoms and socioeconomic variables also impacted ratings of satisfaction with provider communication and trust in our LS population. Our finding regarding higher levels of education being associated with higher provider satisfaction is contrary to what has been reported in the general oncology literature assessing overall satisfaction with health care. Specifically, higher education has been associated with lower levels of satisfaction 44, 45. However, we also found evidence supporting increased provider satisfaction for individuals who self‐reported financial concerns. Given that specific qualities such as physician skill, provider communication, and physician contact with patients are key components of overall satisfaction with health care, our contradictory results for these socioeconomic variables in the LS population merit additional research 44, 45. While we found no significant predictors for the interpersonal or comprehensive subscale measures, prior research with cancer survivors has shown that the comprehensive aspects of the provider relationship (feeling like the provider knows you as a whole person) as well as higher levels of perceived patient‐centeredness are key factors in overall provider satisfaction 19. One of the primary reasons that we were interested in examining factors that predict provider satisfaction in the LS population is because we know that provider recommendations and provider satisfaction play key roles in patient adherence to screening and surveillance guidelines and that nonadherence can be life‐threatening 16, 17, 18, 19, 20. While we have identified some factors that influence provider satisfaction in LS CRC survivors, further research is needed in this unique population, particularly in relation to interpersonal and comprehensive aspects of care.

For sporadic CRC survivors, older age, higher anxiety, and healthcare utilization impacted healthcare provider satisfaction. Specifically, older survivors reported lower levels of satisfaction with respect to provider communication and the interpersonal aspects of their care. CRC survivors who reported higher anxiety also reported lower levels of satisfaction with patient–provider communication. Additionally, the more often a patient sees a HCP, the more satisfied he or she is with how much knowledge that HCP has about him or her as a person. This finding aligns with prior research showing that cancer survivors who are able to ask their providers questions and have their providers explain things in a way they can understand are more satisfied with their care 46.

Taken together, our study findings provide information that is relevant to both clinicians and researchers in regard to factors that influence provider satisfaction in CRC survivors both with and without LS and contribute to the small body of literature that exists on provider satisfaction in an oncology setting. We found evidence to suggest that patients with LS, particularly those who are being treated outside of CCCs, are less satisfied with their HCP than sporadic CRC survivors; however, many of the factors that we hypothesized might predict provider satisfaction in this population were not significant in our regression models. Specifically, we expected that both healthcare experiences and psychosocial factors would have a greater impact on patient satisfaction with their HCPs. While some of these variables were significant in our models, the extent was less than expected based on the literature. Despite these important conclusions and comparisons between LS and sporadic CRC survivors, we recognize that our study is limited by the small sample sizes of both groups as well as the potential bias in recruitment from two different pools of patients with LS. Recruitment for this hard‐to‐reach hereditary cancer population as well as case–case matching was painstakingly undertaken to ensure high‐quality data. Additionally, the control sample is drawn from one CCC, limiting our ability to assess location of care in the sporadic CRC population. One other study limitation is the lack of data on provider specialties as participants were asked to rate the provider most involved in coordinating his/her care, but were not asked to identify specialty areas. This data should be collected in future studies. Given the above‐mentioned factors, the results from this study may not be generalizable to all CRC survivors with or without LS.

We believe that the findings from this study contribute to the scant literature on patient satisfaction with their HCPs both in the oncology literature generally and in the LS population specifically. We discovered a gap in patient satisfaction with their HCPs in these two patient populations that had not previously been demonstrated in the literature and also identified predictors of provider satisfaction that are unique to each of these CRC survivor populations. We also documented that location of care is a key factor in determining patient satisfaction with their HCPs for LS CRC survivors. While these findings can aid HCPs as well as researchers, we believe that further research is needed in a larger sample as well as with qualitative data to more closely examine characteristics of the patient–provider relationship and to identify additional predictors of patient satisfaction with HCPs. This work also needs to be expanded to assess the potential impact of patient satisfaction with their HCPs on adherence to medical management recommendations in LS CRC survivors, particularly those receiving care outside of CCCs.

## Conflict of Interest

The authors declare no conflict of interests.

## Supporting information


**Table S1.** Comparison of LS and sporadic CRC survivors on study variables.
