# Supplementary material for: A comparison between Lynch syndrome and sporadic colorectal cancer survivors’ satisfaction with their healthcare providers
Source: Cancer Med. 2017 Feb 17;6(3):698–707. doi: 10.1002/cam4.1033 (PMC5345658; doi:10.1002/cam4.1033)
Supplement: Supplementary file 1 — Table S1. Comparison of LS and sporadic CRC survivors on study variables. [file CAM4-6-698-s001.docx]

| **Characteristic** | | **Difference between recruitment source**  **(Chi-Square Probability)** |
| --- | --- | --- |
| **Mean Age (SD)** | | 0.4786 |
| **Gender** | Female | 0.6091 |
|  | Male |  |
| **Marital Status** | Married | 0.7151 |
|  | Not Married | 0.9856 |
| **Race** | White | 0.2702 |
|  | Non White |  |
| **Child** | Have Children | 0.5900 |
|  | No Children |  |
| **Work** | Working Full or Part Time | 0.2699 |
|  | Not Working |  |
| **Education** | Less than College | 0.2699 |
|  | College Degree | 0.2699 |
|  | Post Graduate | 0.2249 |
| **Financial Situation** | Financial Difficulty | 0.7876 |
|  | No spare money | 0.4495 |
|  | Can afford special things | 0.7327 |
| **Location of Treatment** | Comprehensive Cancer Centers | N/A |
|  | Non-Comprehensive Cancer Centers | N/A |
| **Health Care Experiences (Number)** | Doctor visits (past 6 months) | 0.1199 |
|  | Emergency Room visits | 0.4870 |
|  | Different Hospital Stays | 0.2679 |
|  | Total Overnight Hospital stays | 0.3308 |
| **Psychosocial Metrics** | CES-D Scale Score | 0.4112 |
|  | TRAIT Score | 0.7325 |
|  | STATE Score | 0.5062 |
| **Social Support Scales** | Krause Social Support Satisfaction Scale | 0.1967 |
|  | Lubben Social Support Family Scale | 0.9213 |
|  | Lubben Social Support Friend Scale | 0.2995 |

**Supplementary Table 1. Comparison of LS and Sporadic CRC Survivors on Study Variables**
